# Brucella Melitensis As Causative Agent for Neck Abscess in an Endemic Area

**DOI:** 10.4274/balkanmedj.2015.1143

**Published:** 2017-01-05

**Authors:** Styliani Sarrou, Charalampos Skoulakis, Jiannis Hajiioannou, Efi Petinaki, Ioannis Bizakis

**Affiliations:** 1 Department of Microbiology, University Hospital of Larissa, Larissa, Greece; 2 Department of Otorhinolaryngology, University Hospital of Larissa, Larissa, Greece

**Keywords:** Brucella melitensis, neck abscess, molecular microbiology

## Abstract

**Background:**

Brucellosis, a zoonotic disease, is very common in the Mediterranean basin and a major concern in livestock areas. We present a rare case of a *Brucella*-caused abscess in the neck of a stock-breeder in an endemic Greek area.

**Case Report:**

A 39-year-old male, living in the rural area of Thessaly, presented with a mass in the left area of his neck. Clinical examination and imaging tests revealed an abscess in the left sternocleidomastoid muscle. Sampling of the abscess by fine-needle aspiration yielded inflammatory fluid (17x10^3^ cells/μL). Molecular sequencing (16S rRNA polymerase chain reaction) performed directly in the clinical sample identified the presence of *Brucella melitensis* within 24 hours after material sampling. The microorganism was isolated in agar media four days later. The Rose-Bengal test was negative, while the Brucellacapt test showed titer 1/320. Given the results obtained with these molecular techniques, the patient was offered treatment with streptomycin (1 g for 3 weeks) and oral doxycycline (100 mg twice daily for 6 weeks), concurrently.

**Conclusion:**

In areas endemic for brucellosis, the investigation of a patient with a neck abscess should include *Brucella* spp. among possible causative agents.

Brucellosis is a bacterial infection caused by *Brucella* spp., which spreads from animals to humans, most often via unpasteurized milk, cheese and other dairy products. More rarely, the microorganism can be spread through the air or through direct contact with infected animals. The disease is very common in the Mediterranean countries with Greece, Spain, Italy and Portugal accounting for the 73% of the total number of confirmed brucellosis cases in Europe (1). In Greece the incidence of brucellosis is 1.11 cases/100.000 inhabitants; however in the area of Thessaly in central Greece the rate rises to 8.03 cases/100.000 inhabitants (2). Thessaly is a rural area with 1.200.000 inhabitants, while, the estimated number of goats and sheep in the region is above 2 million. Despite the efforts of local authorities to vaccinate the herds, the disease remains endemic.

Brucellosis symptoms may include fever, weakness, malaise, headache, anorexia, joint pain, constipation, rigors, splenomegaly, cough, sore throat and abdominal tenderness (3). In addition, several cases of abscesses due to this microorganism have been published, (4,5,6). In this case report, we present a rare case of a neck abscess caused by *Brucella melitensis* in a stock breeder from Thessaly.

## CASE PRESENTATION

A 39-year-old man was admitted to the department of otorhinolaryngology with a mass, which had developed within last 15 days, in the left anterior sternocleidomastoid muscle region. He complained of episodes of fever reaching 38 oC. The patient noted that the mass was progressively growing and painful. He lived and had been working as stockbreeder in Thessaly while his rest medical history was unremarkable.

**Figure 1:**
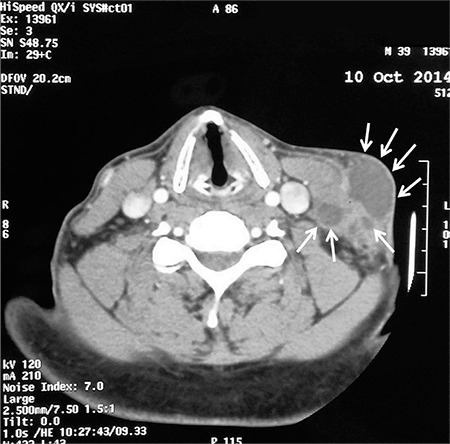
Axial CT scan of the neck shows multiple cystic lesions on the left neck compatible with an abscess.

Physical examination revealed a fluctuating, 2.5x4.5-cm tender mass in his neck. Computed tomography (CT) of the neck with intravenous contrast showed an approximately 3x1.8x5 cm, well defined mass located anterior to the left sternocleidomastoid muscle and two other smaller lesions approximately 1 cm deeper. These findings were suggestive of an abscess formation (Figure 1).

Blood count results revealed hemoglobin of 15.9 g/dL, a white blood cell count of: 9x10^3^/per microliter (neutrophils: 70%, lymphocytes 22%, monocytes 8%) and a platelet count of 325x10^3^ per microliter. The erythrocyte sedimentation rate was 15 mm/h and C-reactive protein: 0.4 mg/dL (normal value <0.5 mg/dL). The results of biochemical analysis were within normal limits.

Sampling from the abscess was performed by ultrasound (US)-guided fine-needle aspiration (FNA). The aspirate was immediately sent to pathological and microbiological laboratories for analysis. In addition, blood aerobic and anaerobic cultures (BD, BACTEK) were also taken on his admission.

According to the FNA results, the lesion was found to involve the lymph node. The microscopic diagnosis revealed granulomatous lymphadenitis with necrosis. The microbiological analysis included firstly cell counting that showed inflamed material of the abscess with 17x10^3^ cells/per microliter of which 70% were neutrophils. In addition, Gram and Ziehl-Neelsen (Z-N) stains, cultures for common bacteria and mycobacteria and a broad-range 16S rRNA PCR able to detect and to identify bacterial DNA directly in the clinical sample, were also performed (7). Both Gram and Z-N stains were negative for detection of any bacteria and mycobacteria, while 16S rRNA PCR revealed the presence of *Brucella* spp. within 24 hours from the time of aspiration of the sample. After 4-days incubation, a Gram negative coccobacillus, positive for catalase, oxidase and urease, was isolated in cultures of the aspirate, confirming the result of molecular methods. Identification of *Brucella melitensis* was established by PCR using specific primers after DNA extraction from the colonies (8). We note that blood cultures obtained from the patient remained sterile even after prolonged incubation (20 days).

Soon after the results of 16S rRNA polymerase chain reaction (PCR) were obtained, serum was taken from the patient for Rose-Bengal (Omega Diagnostics) and Brucellacapt (Vircell SL; Granada, Spain) testing. The Rose-Bengal test was negative, while the Brucellacapt test revealed a titer of 1/320. Notably, in Thessaly, this titer is interpreted as an uncertain result due to the high exposure of the population to *Brucella* (9).

The patient could not be submitted to an magnetic resonance imaging scan because he was claustrophobic and refused to undergo sedation. The results of the CT scan suggested that the patient did not have cervical spondylodiscitis. We therefore decided to treat the patient with a combination of streptomycin 1 g for 3 weeks and oral doxycycline 100 mg twice daily for 6 weeks. If the patient had been diagnosed with spondylodiscitis, the dosage would have been tripled (streptomycin, doxycycline and rifampicin) for at least 12 weeks. Three days after his admission in our hospital the abscess was drained automatically and fully resolved until the conclusion of the antibiotic treatment. After a further 8 month of follow up, including clinical examination and serological tests, he had no evidence of recurrence.

Written informed consent was obtained from the patient for the publication of this case report and accompanying images.

## DISCUSSION

The differential diagnosis of a neck mass in adults includes three main causes: congenital anomalies, neoplasms and infections. Comprehensive medical history combined with clinical examination and aided by medical imaging lead to accurate diagnosis. In our case medical history and clinical examination suggested the inflammatory nature of the lesion, confirmed by CT scan. The identification of the causative agent is of paramount importance for the proper treatment. The most common infectious causes of abscess formation (bacterial and viral) include *Staphylococcus* and *Streptococcus* species, *Mycobacterium tuberculosis* and atypical mycobacterial infections, *Bartonella henselae, Toxoplasma gondii, Epstein-Barr* virus and *Actinomyces israelii*. *Brucella* spp. remains an unusual pathogen for a neck abscess.

After ingestion or inoculation, *Brucella* species invade the mucosa, where polymorphonuclear leukocytes and activated macrophages mediate immune responses to eradicate the bacteria. However, the microorganism can multiply and survive intra-cellularly by inhibiting and counteracting bactericidal effects within the phagosome. They are then transported intra-cellularly via the lymphatics to organs rich in reticuloendothelial cells, and from there travel to other organs and tissues, where they can cause inflammation, granuloma formation, necrosis, and abscess formation. Many reports have shown abscess formation in vertebral arch, in spleen, in liver, in renal parenchyma etc. due to hematogenous dissemination (4,5,6). A thorough Medline (PubMed) search revealed only one case with neck abscess caused by *Brucella melitensis* (10). Interesting finding in this case as in ours, was the fact that both patients did not manifest systemic symptoms and findings (weight loss, night sweats, fatigue, severe malaise, fever etc). Although the most frequent reason for neck abscess is spreading through hematogenous way, the exact way of spreading has not been elucidated. However, we could speculate that the patient, who is a breeder, had probably inoculated the microorganism in his neck by direct contact with secretions of infected animals.

Brucellosis may present as a multi-systemic disease involving various organs, as septicemia, as chronic disease with atypical symptoms or as a focal form without systematic findings. Thus, the variety of the appearing symptoms renders clinical diagnosis quite challenging. Our patient did not complain of any major systemic symptoms apart from fever and there were no findings other than the neck mass. In such cases the contribution of the laboratory test in diagnosis is of great value. Despite the use of various serological tests as diagnostic tool for brucellosis, sometimes these tests lack sensitivity and specificity. On the other hand, *Brucella* spp. is a slow-growing microorganism that takes long time to be cultured in agar media. The limitations of these conventional microbiological methods, especially in focal forms of the disease, necessitate molecular assays for the rapid diagnosis of Brucellosis. In clinical settings, where PCR assays are not available, a prolonged incubation of cultures (more than a week) could improve the detection of the microorganism.

In conclusion, in areas endemic for brucellosis, the evaluation of a patient with a neck mass should include *Brucella* spp. among the possible causative agents.
